# Miscibility Studies of Hyaluronic Acid and Poly(Vinyl Alcohol) Blends in Various Solvents

**DOI:** 10.3390/ma13214750

**Published:** 2020-10-23

**Authors:** Katarzyna Lewandowska

**Affiliations:** Faculty of Chemistry, Nicolaus Copernicus University in Toruń, Gagarin 7, 87-100 Toruń, Poland; reol@umk.pl

**Keywords:** polymer blends, poly(vinyl alcohol), hyaluronic acid, rheological properties

## Abstract

In this study, blends based on hyaluronic acid (HA) and poly(vinyl alcohol) (PVA) were characterized by the viscometric method, steady shear rheological tests and FTIR spectroscopy (Fourier Transform Infrared Spectroscopy). Viscometric studies showed the miscibility of HA and PVA in distilled water: 0.1 mol dm^−3^ NaCl and 0.1 mol dm^−3^ HCl. The method proposed by Garcia et al. was applied to assess the miscibility of polymers, while *Δ[η]* and *Δb* were introduced to determine of miscibility from the Huggins plots. The viscometric data showed that the attractive forces of HA and PVA were dominant when dissolved in 0.1 mol dm^−3^ NaCl and 0.1 mol dm^−3^ HCl, while, in distilled water, repulsive forces played the leading role. All polymer solutions were well characterized using a power law model, and exhibited non-Newtonian behavior with pseudoplasticity increasing with the increasing weight fraction of HA in 0.1 mol dm^−3^ NaCl and 0.1 mol dm^−3^ HCl. FTIR studies exhibited the formation of new intermolecular interactions between HA and PVA via hydrogen bonding.

## 1. Introduction

Hyaluronic acid (HA) is a natural macromolecule isolated from natural sources such as bacteria (*Streptococcus equi*), rooster combs and others [1,2]. It is a class of the nonsulfated glycosaminoglycan, and consists of repeating disaccharide units of D-glucuronic acid and N-acetyl glucosamine linked by β(1,4) and β(1,3) glucosidic bonds [1,2,3,4]. HA possesses interesting properties such as biodegradability, non-toxicity for living organisms, solubility in aqueous solutions, bioactivity and biocompatibility, which have resulted in increased interest in the material’s potential medical and cosmetic uses, e.g., in the fields of tissue engineering, applications in wound healing, drug delivery, artificial skin, and as an ingredient in cosmetics that provides anti-aging and moisture-supplying effects to the skin [3,5,6,7,8,9,10,11,12,13]. The drawbacks of using HA as a biomaterial is its rapid degradation and poor mechanical stability [10]. There are several types of processes that can improve the polymeric structure and properties of HA. One such method involves mixing with other components such as proteins, polysaccharides, synthetic polymers, hydroxyapatite, nanofillers and inorganic and/or organic compounds [10,14,15].

In this study, blends composed of HA with poly(vinyl alcohol) (PVA) at different ingredient ratios have been prepared as materials designed for biomedical and cosmetic applications. PVA is a water-soluble, non-ionic synthetic polymer. Its ease of preparation, as well as good biocompatibility, biodegradability and physiological inactivity, promoted the widespread use of PVA in diverse areas such as food, drug delivery systems, cosmetics and other medical and industrial applications [16,17,18,19,20]. Therefore, HA properties can be improved by blending hyaluronic acid with PVA. These blends can be used to produce implants, wound dressings and in drug delivery [13,21]. Additionally, the viscosity behavior of polymers and their blends are essential for many applications. In the case of cosmetic and food usage, viscosity behavior at different temperatures is essential in order to design proper formulations and check their applicability under various conditions. Based on rheological studies, one can design a product with tailor-made properties for specific uses, for example, tissue engineering purposes.

Several reports examined HA blends with PVA as films [21], gels [22,23,24], cryo-gel membranes [25,26,27] and blends based on HA and poly(vinyl alcohol)-borax association (PVAs) [28]. They showed that the chemically cross-linked PVA/HA hydrogels can be attractive materials for tissue engineering [22]. In the case of HA/PVA cryo-gels, modification of PVA hydrogels is achieved through the addition of HA which enhances the properties and bioactivity of films, compared with pure PVA hydrogel [25].

The purpose of our study was to investigate the influence of various solvents on the physical properties of HA/PVA blends. The miscibility, structure and rheological properties were characterized based on the viscometric technique, steady shear rheological measurements and FTIR spectroscopy. Miscibility is an important aspect for the preparation of new materials based on the blending of two polymers. The properties of these materials depend on the specific interactions between the ingredients. If such interactions are possible, the blending of chemically different polymer components can occur even at the molecular level, and the properties of the blend can exhibit a synergistic effect. The estimation of the miscibility in polymer blend solutions comprising a common solvent for polymer A and B relies on the comparison of the experimental interaction parameters with ideal values. Reports have shown that a positive deviation between the experimental and ideal values indicates a miscible system in which attractive forces are dominant [15,19,29], whereas negative deviation indicates an immiscible system in which repulsive forces play a leading role. To the best of the author’s knowledge, the miscibility of HA/PVA in various solvents by the viscometric method has yet to be investigated. The viscometric method is a simple, quick and inexpensive evaluation approach for the miscibility of polymers in solution.

## 2. Materials and Methods

### 2.1. Materials

Unless otherwise stated, all materials, chemicals and reagents used in this work were supplied by Sigma-Aldrich (Poznan, Poland), Chempur (Piekary Śląskie, Poland) and POCh (Avantor, Gliwice, Poland). Poly(vinyl alcohol) with a 99% degree of hydrolysis (DH) and an average molecular weight viscosity of 130,000 was used. HA powder with an average molecular weight viscosity of 8000 was used. All chemicals were of analytical grade and used as received without further purification.

Preparation of polymer solutions and thin films consisted of the polymer sample being dissolved in distilled water: 0.1 mol dm^−3^ NaCl and 0.1 mol dm^−3^ HCl. The solutions of HA and PVA were mixed at different proportions (75/25, 50/50 and 25/75). Thin films were obtained by the solution casting method. The polymer and blend solutions were poured onto a glass plate, covered with polyethylene film and evaporated at room temperature (298 K) for 24 h. The films were then dried at 323 K for 24 h under a vacuum and stored in a desiccator. All films had a similar thickness of approx. 20 μm.

### 2.2. Viscometric Studies

Viscometric studies of diluted solutions (*c* < 1.5%) were conducted at 25 ± 0.1 °C using an Ubbelohde capillary viscometer in a controlled thermostatic bath. Each stock polymer solution with known weight was added to the viscometer and flow time was measured at constant temperature within ±0.1 °C. A certain amount of solvent was added to the solution successively to obtain a concentration gradient (5 various concentrations). Then, the flow time of the solvent was measured. The flow time of each solution was taken as the average of several readings. The specific viscosity of dilute polymer solution was calculated by Equation (1):

(1) $$\eta_{sp}=\frac{t-t_{0}}{t_{0}}$$

where *t* is the flow time of the polymer solution and *t_0_* is the flow time of the solvent.

The values of the miscibility parameter and intrinsic viscosity were calculated using the Huggins equation from solutions of several concentrations [30,31]. The miscibility was evaluated by a comparison of the experimental and ideal values of *b_m_* and *[η]_m_*. The values of miscibility parameters were obtained using the same methods as described in [29]. In previous papers [29,32], the main aspects of the use of the method proposed by Garcia et al. [33] to investigate of miscibility in dilute solutions are described. Briefly, the experimental interaction parameter was calculated using Equation (2):

(2) $$\frac{{\left(\eta_{sp}\right)}_{m}}{c_{m}}={\left[\eta \right]}_{m}^{exp}+b_{m}^{exp}c_{m}$$

where *(η_sp_)_m_* is the specific viscosity of the polymer blend (dimensionless), *(_sp_)_m_/c_m_* is the reduced viscosity (dL/g), *c_m_* is the total concentration of the polymer blend (g/dL), ${\left[\eta \right]}_{m}^{exp}$ is the experimental intrinsic viscosity (dL/g) and $b_{m}^{exp}$ is the experimental interaction parameter (dL/g)^2^. The slope and intercept of the graph (the reduced viscosity (*η_sp_/c*) vs. total concentration (*c*)) gave the values of $b_{m}^{exp}$ and ${\left[\eta \right]}_{m}^{exp}$, respectively. The ideal interaction parameter was described by Equation (3):

(3) $$b_{m}^{id}=w_{A}b_{A}^{2}+w_{B}b_{B}^{2}$$

where $b_{m}^{id}$ is the ideal interaction parameter (dL/g)^2^,$\;w_{A}$ and $w_{B}$ are the weight fractions of polymers A and B, respectively, *b_A_* and *b_B_* are the interaction parameters of each individual polymer. The miscibility criterion is as follows:

if $\Delta b_{m}=b_{m}^{exp}-b_{m}^{id}>0$ the polymer blend is miscible,

if $\Delta b_{m}=b_{m}^{exp}-b_{m}^{id}<0$ the polymer blend is immiscible.

According to Garcia’s method [33], another criterion based on the experimental and ideal values of the intrinsic viscosity (*[η]_m_*) was proposed. The experimental value (${\left[\eta \right]}_{m}^{exp}$) was estimated from the intercept of the graph according to Equation (2). The ${\left[\eta \right]}_{m}^{id}$ value was described by Equation (4):

(4) $${\left[\eta \right]}_{m}^{id}=w_{A}{\left[\eta \right]}_{A}+w_{B}{\left[\eta \right]}_{B}$$

where *[η]_A_* and *[η]_B_* are the intrinsic viscosity (dL/g) of polymers A and B, respectively. Thus, if *Δ[η]* < 0, the polymer blend is miscible, and if *Δ[η]* > 0, the polymer blend is immiscible.

### 2.3. Steady Shear Measurements

Rheological measurements of polymer solutions were conducted using a Bohlin Visco 88 rotary viscometer (Malvern Panalytical, Malvern, UK) equipped with concentric cylinders. Apparent shear viscosity was recorded as a function of shear rate from 20 to 1230 s^−1^ and at various temperatures of 25 °C, 30 °C, 35 °C and 40 °C. All solutions were equilibrated at constant temperature for 15 min before rheological tests. The power law model (Equation (5)) was used to fit the experimental viscosity curves [34,35]:

(5) $$\eta_{a}=k{\dot{\gamma }}^{n-1}$$

In Equation (5), *k* and *n* are constants, *k* is the consistency index (Pa s^n^) and *n* is the flow behavior index (dimensionless).

### 2.4. FTIR Analysis

IR spectra of the films were obtained with a Nicolet iS10 FTIR spectrophotometer Thermo Fisher Scientific Inc. (Waltham, MA, USA) using Attenuated Total Reflectance (ATR) mode with a diamond as the crystal. Each spectrum was obtained using 64 scans at a resolution of 2 cm^−1^, and a spectral range from 4000 to 600 cm^−1^.

## 3. Results and Discussion

**Figure 1:**
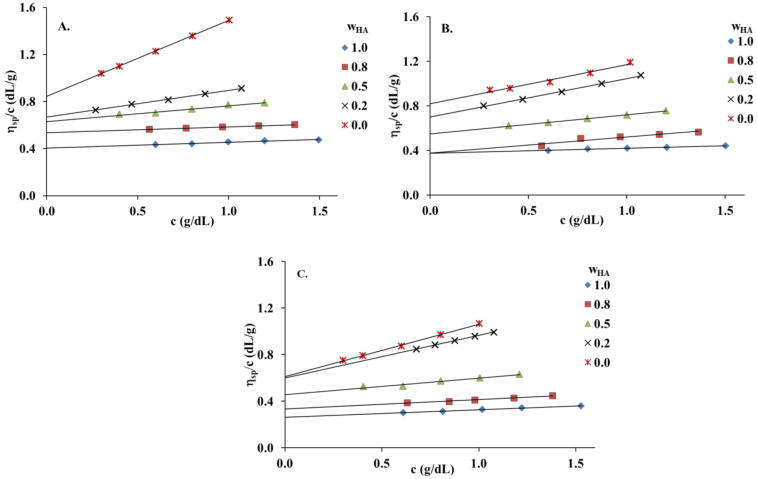
Reduced viscosity (*η_sp_/c*) values of hyaluronic acid (HA), poly(vinyl alcohol) (PVA) and their blends at 25 °C: (**A**) distilled water; (**B**) 0.1 mol dm^−3^ NaCl; (**C**) 0.1 mol dm^−3^ HCl.

**Figure 2:**
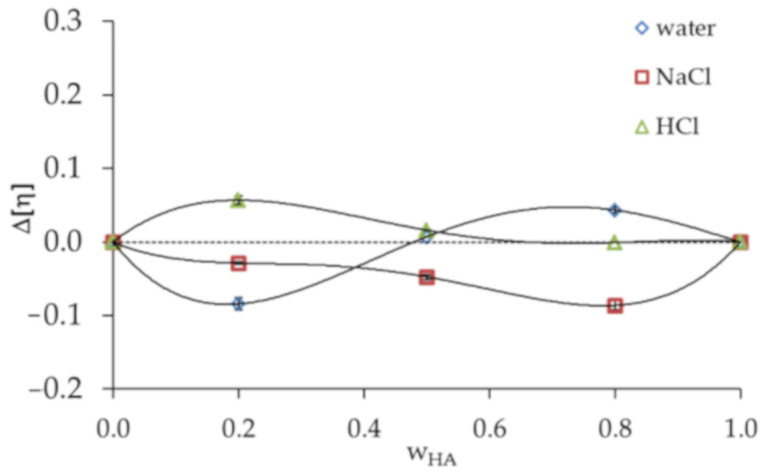
Plot of *Δ[η]* criterion versus HA weight fraction ($w_{HA}$) in HA/PVA blends in various solvents.

Figure 1 presents the Huggins plots (the reduced viscosity (*η_sp_/c*) vs. total concentration (*c*)) for HA and PVA and their blends at different HA weight fractions ($w_{HA}$) in distilled water, 0.1 mol dm^−3^ NaCl and 0.1 mol dm^−3^ HCl. All graphs present linear behavior for native polymers and HA/PVA blends over the entire composition range, suggesting that *[η]* could be established by linear extrapolation to zero concentration. ${\left[\eta \right]}_{n}^{exp}$ and $b_{m}^{exp}$ values were determined using the classical Huggins equation (Equation (2)) [30,31]. The ideal values ($b_{m}^{id}$) were calculated based on Garcia et al. [33]. *Δb_m_* and *Δ[η]* values and comparisons between the different solvents used in polymer systems are tabulated in Table 1 and Figure 2.

The obtained results show that the differences in the *Δb_m_* and *Δ[η]* values in three solvents are significant. The values of the miscibility parameter (*Δb_m_*) are positive for all HA/PVA blends in 0.1 mol dm^−3^ NaCl and 0.1 mol dm^−3^ HCl, distinctly exceeding the range of experimental error. Hence, these blends satisfy the criterion proposed by Garcia et al. [33]. In the case of HA/PVA blends in distilled water, the miscibility parameters are negative or equal zero in the range of experimental errors, indicating the poorer miscibility of this solvent. The observed changes in the miscibility of HA and PVA are related to the polyelectrolyte nature of HA. The addition of NaCl or HCl to the polymer solution causes increased ionic strength in the solution, resulting in reduced repulsive forces (electrostatic interactions) between molecules. Thus, the interaction of HA/PVA blends in 0.1 mol dm^−3^ NaCl and 0.1 mol dm^−3^ HCl mainly consists of hydrogen bonds. In turn, the immiscibility of polymeric components is related to repulsive forces which play a leading role in aqueous solutions without the addition of salt or acid. The greater amount of PVA in the HA solution promotes strong, repulsive interactions between the polymeric components and phase separation in the selected blend system ($w_{HA}$ = 0.2), and is the reason for the negative value of the interaction parameter.

Garcia et al. [33] also proposed the criterion *Δ[η]*; accordingly, all HA/PVA blends in 0.1 mol dm^−3^ NaCl are miscible (Figure 2). In the case of HA/PVA blends in 0.1 mol dm^−3^ HCl, the experimental intrinsic viscosities are higher than ideal values. Attraction between HA and PVA in 0.1 mol dm^−3^ HCl increase the hydrodynamic volume, causing positive deviation. The interaction between the two polymer components in this case is considered to be strong. If the interaction between polymer chains usually predominate over those between the polymer chain and the solvent, the result of attraction is a positive deviation from the ideal value. Interactions between polymer chains might decrease the opportunity for an interaction between the polymer chain and the solvent, weakening the solvation effect and causing the intrinsic viscosity to decrease. Thus, negative deviation is observed [36].

**Figure 3:**
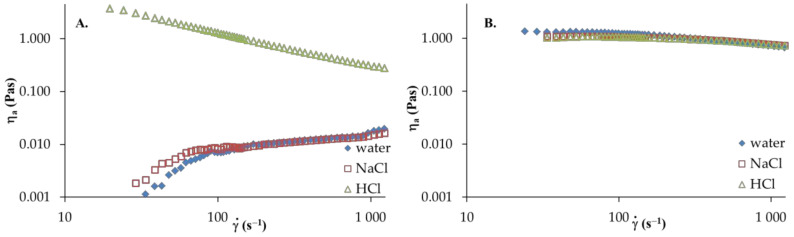
Apparent shear viscosity ${}_{a}$ versus shear rate $\dot{\gamma }$ of HA and PVA: (**A**) HA and (**B**) PVA, T = 25 °C.

**Figure 4:**
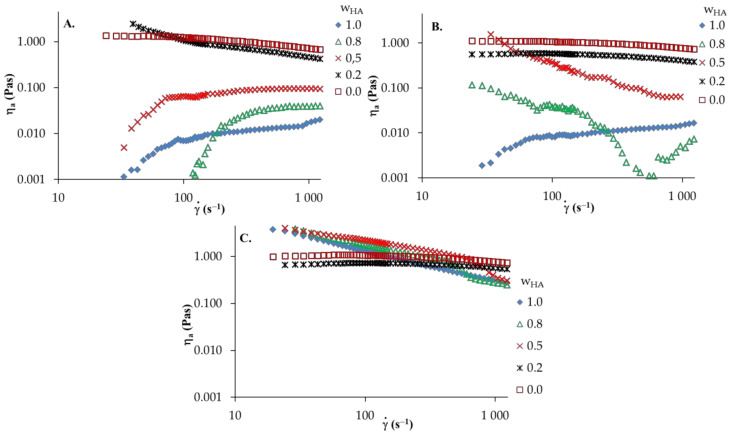
Apparent shear viscosity ${}_{a}$ versus shear rate $\dot{\gamma }$ of HA and PVA and their blends: (**A**) distilled water; (**B**) 0.1 mol dm^−3^ NaCl; (**C**) 0.1 mol dm^−3^ HCl, T = 25 °C, $w_{HA}$—HA weight fraction.

The effect of various solvents on the rheological properties of HA, PVA and their blends were investigated by steady shear measurements. Figure 3 and Figure 4 show the viscosity curves as a function of the shear rate to the polymer samples and HA/PVA blends at 25 °C.

The apparent viscosity of HA solutions in distilled water and 0.1 mol dm^−3^ NaCl (Figure 3A) increase with the increasing shear rate, in which shear-thickening behavior is observed. However, in the aqueous acidic solution of HA, shear-thinning behavior (pseudoplastic nature) is observed; hence, the apparent viscosity decreases with increasing of shear rate. Moreover, the addition of HCl to the aqueous solution causes a marked increase in apparent viscosity. This may be attributed to the strong interactions and complex formations between active HA groups in the network structure and ion pairs with solvent. Furthermore, an increase in shear rate leads to destruction of the network structure and the dispersed HA molecules arrange along the flow direction. Thus, reduced apparent viscosity is observed. For PVA solutions in various solvents, the apparent viscosity of the solution decreases as the shear rate increases, showing a shear-thinning behavior. Moreover, no significant differences in apparent viscosity are observed in any solvent studied. Figure 4 presents the viscosity curves of HA/PVA solutions in various solvents. In HA/PVA blend solutions with $w_{HA}$ ≤ 0.5 in distilled water and 0.1 mol dm^−3^ NaCl as well as in all the blend solutions in 0.1 mol dm^−3^HCl, a shear-thinning behavior is observed (Figure 4). The pseudoplastic effect decreases with increasing PVA content in the blend solutions. Additionally, PVA and HA/PVA blend with $w_{HA}$ = 0.2 (Figure 4C) shows a flow pattern comparable to Newtonian behavior. In the case of the aqueous acidic solution of blends at $w_{HA}$ ≥ 0.5, the apparent viscosity is higher than that for pure polymer solutions, especially for a lower shear rate ($\dot{\gamma }$ < 400s^−1^). This synergy could be explained by the attractive forces between the polymeric components in the blend solutions, promoting increased viscosity values. At a higher shear rate ($\dot{\gamma }$ > 400s^−1^), shear forces destroy the hydrogen bonds that occur in the polymer blends and the observed differences are smaller.

The experimental data were fitted with the power law model (Equation (5)) and the rheological parameters (*n* and *k*) were calculated using a linearized equation. The parameters for polymer solutions and their blends are listed in Table 2. In the case of non-Newtonian fluid (*n* ≠ 1), the viscosity characteristic can be approximately that of the shear-thinning behavior if *n* < 1 and shear-thickening behavior if *n* > 1 [35,37]. Therefore, parameter values for *n* of PVA and HA/PVA blend solutions with $w_{HA}$ ≤ 0.5 in distilled water and 0.1 mol dm^−3^ NaCl as well as all blend solutions in 0.1 mol dm^−3^ HCl are below one, indicating that pseudoplastic behavior becomes more obvious with increasing HA content in the blend solutions. However, the PVA and HA/PVA blend with $w_{HA}$ = 0.2 in 0.1 mol dm^−3^ HCl displays a value of the parameter *n* that is approximate to one (Newtonian flow), especially for a higher temperature (40 °C). In the case of HA and HA/PVA blend solutions with $w_{HA}$ ≥ 0.5 in distilled water and 0.1 mol dm^−3^ NaCl, where the shear thickening effect was observed, the parameter *n* is above one, while, for the aqueous acidic solution of HA, the value of the *n* parameter is below one, indicating stronger shear-thinning property. The obtained low *n* value suggests a high association degree and complex formation between components in solution. The consistency coefficients *k* represent a direct relationship with the apparent viscosity. Therefore, *k* coefficients are expected because the temperature commonly lowers the solution’s viscosity. These observations are consistent with previous reports on HA or PVA solutions [37,38,39,40].

**Figure 5:**
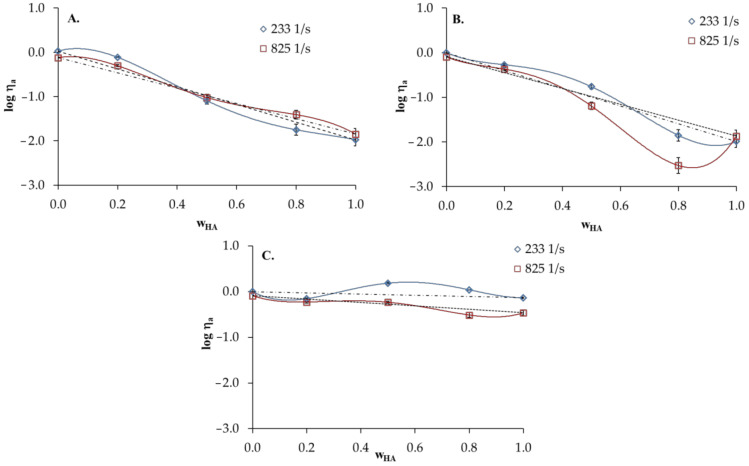
Logarithm of apparent shear viscosity of HA and PVA and their blends versus hyaluronic acid weight fraction ($w_{HA}$) in the blends: (**A**) distilled water; (**B**) 0.1 mol dm^−3^ NaCl; (**C**) 0.1 mol dm^−3^ HCl, T = 25 °C, dotted line—values calculated according to the additivity rule.

Figure 5 presents the logarithm of apparent viscosity (log *η_a_*) for HA/PVA blends vs. HA weight fraction ($w_{HA}$). The experimental log *η_a_* values are drawn together with the values obtained according the additivity rule expressed by Equation (6):

(6) $$\log\eta_{m}=w_{A}\log\eta_{A}+w_{B}\log\eta_{B}$$

where log *η_m_* is the logarithm of the apparent shear viscosity of the polymer blend, log *η_A_* and log *η_B_* are the logarithms of the apparent shear viscosity of polymers A and B, respectively [41].

As shown, the positive deviations from the calculated straight line illustrates the additivity rule of 0.1 mol dm^−3^HCl. In the case of HA/PVA blends in distilled water, log *η_a_* values practically fulfil the linear dependence drawn according to the additivity rule (Figure 5A). Deviations in the log *η_a_* decrease with the increasing shear rate, which is due to the destruction of intermolecular interactions between components under shear forces. HA/PVA blends in 0.1 mol dm^−3^ NaCl display negative deviations in log *η_a_* from the additivity rule. This may indicate that HA and PVA are poorly miscible in 0.1 mol dm^−3^ NaCl.

**Figure 6:**
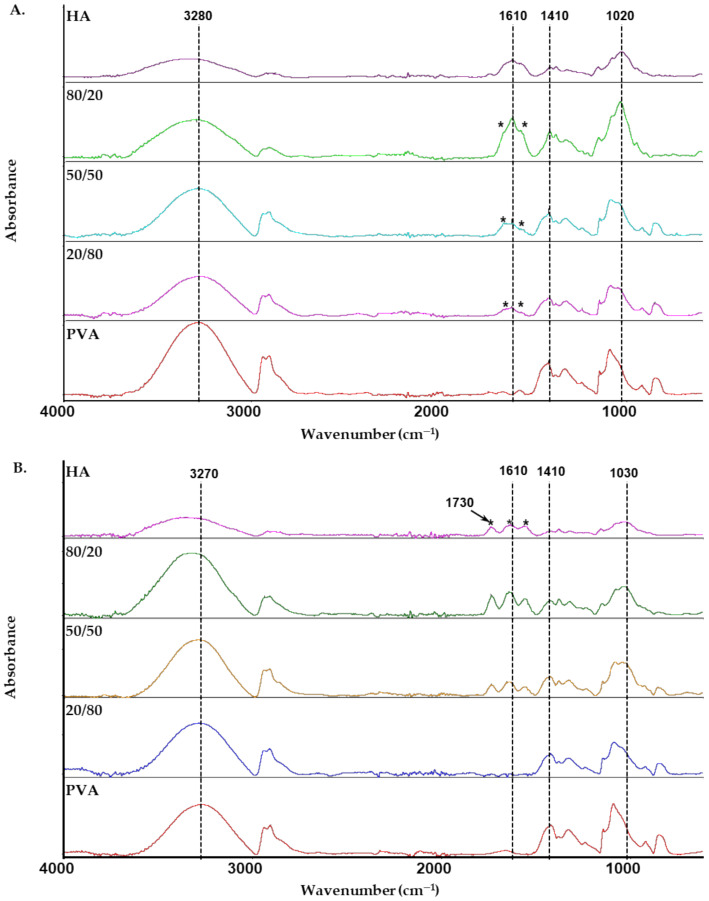
Attenuated Total Reflectance (ATR)–infrared spectra of HA, PVA and their blend films in various solvents: (**A**) distilled water; (**B**) 0.1 mol dm^−3^ HCl.

FTIR is a method widely used to study polymer structures and to detect intermolecular interactions between polymer chains. Figure 6 shows the infrared spectra of pure components and their blends in distilled water and 0.1 mol dm^−3^ HCl. Thus, FTIR allows for changes in the structure of HA/PVA blends, which is dependent on solvent and HA content. The spectrum of HA film was cast from distilled water and shows a broad absorption band at about 3310 cm^−1^ (OH and NH stretching modes); the peak at 1610 cm^−1^ corresponds to carbonyl stretching bands of carboxylate [42,43]. An intense band extending between 900–1200 cm^−1^ corresponds to C-O stretching vibrations in alcohols (1020 cm^−1^ and 1080 cm^−1^), while the shoulder at 1152 cm^−1^ is assigned to C-O-C stretching vibrations in glycosidic groups [41]. In the spectrum of HA film cast from 0.1 mol dm^−3^ HCl solution, amide I and II bands are observed at 1644 cm^−1^ and 1562 cm^−1^ (marked with an asterisk) in which the asymmetric of stretching carboxylate groups greatly decreased. The peak at 1730 cm^−1^ relates to the carbonyl stretching bands of carboxyl groups in HA film [4,42,43]. Therefore, most of the carboxyl groups are protonated in acidic HA solution [42,44,45]. For PVA films, the spectra show the peaks of the stretching vibration of OH groups at 3280cm^−1^ or 3270cm^−1^ and C-O stretching vibrations in the range of 900–1200 cm^−1^.

The interactions occurring in HA/PVA blend films between the components are visible by marked spectral changes, especially in the region of the stretching vibration of OH groups, the C-O stretching vibrations in alcohols and the amide I and II bands. The maximum of the OH stretching band of the HA/PVA blends is located between that corresponding to HA (3340 cm^−1^ or 3310 cm^−1^) and PVA (3280 cm^−1^ or 3270 cm^−1^). The spectra of blend films cast from distilled water show that two sub-bands at approximately 1653 cm^−1^ and 1552 cm^−1^ are intense (marked with an asterisk) and can be attributed to carboxylate groups of HA participating in one or more hydrogen bonds [42,44]. Additionally, the wide band at approximately 1020 cm^−1^ shifts after blending HA with PVA, possibly due to interactions between -COO^-^ and -OH groups of HA and -OH groups of PVA. For blend films cast from 0.1 mol dm^−3^ HCl solution, the spectra of HA/PVA blends with $w_{HA}$ ≥ 0.5 showed bands at 1730 cm^−1^ and 1640 cm^−1^ corresponding to the -COOH groups of HA. These bands are not observed for the blend with low HA content. Thus, the results suggest that other functional groups such as -NHCOCH_3_ can form hydrogen bonds with -OH groups of PVA and create miscible blends. These results are consistent with viscosity and rheological studies which indicate that the blend is miscible. FTIR spectroscopy analysis confirmed the formation of new interactions between the HA and PVA which influenced the viscosity behavior and observed synergistic effects.

## 4. Conclusions

Herein, the impact of the solvent on the miscibility of HA/PVA blends using the viscometric method, steady shear rheological measurements and FTIR analysis have been investigated. It was found that solvent has a large effect on the viscosity behavior. HA/PVA blends are completely miscible due to the attractive forces through hydrogen bonding in 0.1 mol dm^−3^ NaCl and 0.1 mol dm^−3^ HCl. The miscibility of HA with PVA is related to HA conformation changes and interactions driven by hydrogen bonding between -COOH and -NHCOCH_3_ groups in HA and -OH groups in PVA. Analyses of the rheological properties show that the HA/PVA blends at $w_{HA}$ ≥ 0.5 in 0.1 mol dm^−3^ HCl exhibit a larger apparent viscosity than native polymers. Therefore, interactions between HA and PVA and positive synergistic effects that occur in aqueous acidic solutions are significant. HA and PVA are potential candidates for the preparation of new functional materials. Additionally, they play a key role in the properties of the resulting polymer blends for different applications, including tissue engineering, wound dressings, drug delivery systems, and cosmetics.

## Figures and Tables

**Table 1:** Experimental and ideal miscibility parameters *b_m_* of HA/PVA blends.

$w_{HA}$	$b_{m}^{exp}\pm SD{\;}^{1}$ **(dL/g)^2^**	$b_{m}^{id}\pm SD$ **(dL/g)^2^**	$\Delta b_{m}^{}$	**Remarks**
Solvent: Distilled Water				
0.2	0.228 ± 0.021	0.292 ± 0.021	−0.0640	immiscible
0.5	0.133 ± 0.021	0.125 ± 0.021	0.0080	miscible
0.8	0.0502 ± 0.021	0.0492 ± 0.021	0.0010	miscible
Solvent: 0.1 NaCl mol⋅dm^−3^				
0.2	0.345 ± 0.021	0.226 ± 0.021	0.119	miscible
0.5	0.175 ± 0.021	0.0989 ± 0.021	0.0711	miscible
0.8	0.143 ± 0.021	0.0428 ± 0.021	0.100	miscible
Solvent: 0.1 HCl mol⋅dm^−3^				
0.2	0.367 ± 0.021	0.291 ± 0.021	0.0760	miscible
0.5	0.143 ± 0.021	0.129 ± 0.021	0.0140	miscible
0.8	0.0810 ± 0.021	0.0587 ± 0.021	0.0223	miscible

^1^ SD—standard deviation.

**Table 2:** The rheological parameters from power law model for HA, PVA and their blends as functions of temperature.

$w_{HA}$	**T (°C)**	***n***	***k*** **(Pas)^n^**	**R^2^**
Distilled Water				
0.0	25	0.79	3.29	0.997
-	40	0.82	3.15	0.999
0.2	25	0.60	7.06	0.987
-	40	0.59	5.45	0.976
0.5	25	1.19	2.66 × 10^−2^	0.997
-	40	1.63	7.82 × 10^−4^	0.954
0.8	25	1.64	5.66 × 10^−4^	0.963
-	40	2.09	1.38 × 10^−5^	0.965
1.0	25	1.43	9.29 × 10^−4^	0.990
-	40	1.70	1.19 × 10^−4^	0.994
0.1 mol dm^−3^ NaCl				
0.0	25	0.90	1.64	0.997
-	40	1.01	0.448	0.995
0.2	25	0.89	0.909	0.997
-	40	0.97	0.164	0.995
0.5	25	0.23	9.85 × 10^−2^	0.816
-	40	0.30	3.98 × 10^−3^	0.935
0.8	25	0.29	1.62	0.991
-	40	0.18	2.13	0.765
1.0	25	1.27	2.37 × 10^−3^	0.998
-	40	1.51	3.55 × 10^−4^	0.999
0.1 mol dm^−3^ HCl				
0.0	25	0.91	1.53	0.997
-	40	1.02	0.493	0.997
0.2	25	0.96	0.848	0.995
-	40	1.02	0.237	0.998
0.5	25	0.56	16.7	0.997
-	40	0.47	14.7	0.999
0.8	25	0.40	29.2	0.994
-	40	0.39	16.2	0.999
1.0	25	0.35	25.4	0.995
-	40	0.40	15.5	0.999
